# Cognitive-behavioral therapy vs. light therapy for preventing winter depression recurrence: study protocol for a randomized controlled trial

**DOI:** 10.1186/1745-6215-14-82

**Published:** 2013-03-21

**Authors:** Kelly J Rohan, Maggie Evans, Jennifer N Mahon, Lilya Sitnikov, Sheau-Yan Ho, Yael I Nillni, Teodor T Postolache, Pamela M Vacek

**Affiliations:** 1 Department of Psychology, University of Vermont, John Dewey Hall, 2 Colchester Avenue, Burlington, VT, 05405-0134, USA; 2 Mood and Anxiety Program, Department of Psychiatry, University of Maryland School of Medicine, Baltimore, MD, USA; 3 Department of Medical Biostatistics, University of Vermont College of Medicine, Burlington, VT, USA

**Keywords:** Seasonal affective disorder, Clinical trial, Cognitive-behavioral therapy, Light therapy, Depression recurrence, Prevention

## Abstract

**Background:**

Seasonal affective disorder (SAD) is a subtype of recurrent depression involving major depressive episodes during the fall and/or winter months that remit in the spring. The central public health challenge in the management of SAD is prevention of winter depression recurrence. Light therapy (LT) is the established and best available acute SAD treatment. However, long-term compliance with daily LT from first symptom through spontaneous springtime remission every fall/winter season is poor. Time-limited alternative treatments with effects that endure beyond the cessation of acute treatment are needed to prevent the annual recurrence of SAD.

**Methods/design:**

This is an NIMH-funded R01-level randomized clinical trial to test the efficacy of a novel, SAD-tailored cognitive-behavioral group therapy (CBT) against LT in a head-to-head comparison on next winter outcomes. This project is designed to test for a clinically meaningful difference between CBT and LT on depression recurrence in the next winter (the primary outcome). This is a concurrent two-arm study that will randomize 160 currently symptomatic community adults with major depression, recurrent with seasonal pattern, to CBT or LT. After 6 weeks of treatment in the initial winter, participants are followed in the subsequent summer, the next winter, and two winters later. Key methodological issues surround timing study procedures for a predictably recurrent and time-limited disorder with a focus on long-term outcomes.

**Discussion:**

The chosen design answers the primary question of whether prior exposure to CBT is associated with a substantially lower likelihood of depression recurrence the next winter than LT. This design does not test the relative contributions of the cognitive-behavioral treatment components vs. nonspecific factors to CBT’s outcomes and is not adequately powered to test for differences or equivalence between cells at treatment endpoint. Alternative designs addressing these limitations would have required more patients, increased costs, and reduced power to detect a difference in the primary outcome.

**Trial registration:**

Clinicaltrials.gov identifier NCT01714050

## Background

Winter seasonal affective disorder (SAD) is a subtype of recurrent depression involving major depressive episodes during the fall and/or winter months that remit in the spring [1]. SAD prevalence increases with latitude in the US, ranging from 1.4% in Florida to 9.9% in Alaska [2,3]. Averaging across latitude, SAD affects an estimated 5% of the US population [4]—over 14.5 million Americans. Untreated SAD-related major depressive episodes last, on average, about 5 months of the year before spontaneous springtime remission (personal communication, Norman E. Rosenthal, MD, May 2005) and are associated with significant impairments in overall health, emotional well-being, and daily and social activities [5]. In a multi-site study including over a thousand SAD patients, participants reported a mean age for onset of 27.2 years and, on average, 13.4 past fall/winter major depressive episodes [6]. Taken cumulatively, these data suggest that SAD patients spend over 40% of the year struggling with substantial depressive symptoms during most years, beginning in young adulthood.

As a first-line SAD treatment, clinical practice guidelines recommend daily light therapy (LT) from onset of first symptom through spontaneous springtime remission each fall/winter season [7]. LT is the established and best available acute SAD treatment. A recent quantitative analysis of trials comparing LT to credible controls concluded that LT is associated with significant reductions in depression severity for SAD in the initial winter [8]. A pooled analysis of LT studies [9] concluded that 53% of individuals with SAD overall and 43% of moderate to severe SAD cases meet remission criteria by the end of an LT trial.

LT, like most psychiatric treatments, is a palliative treatment that needs to be continued with regularity over time to prevent relapse and recurrence; however, available data suggest that the majority of SAD patients are not willing/able to comply with the treatment over subsequent fall/winter seasons. A retrospective follow-up survey of SAD patients treated with LT at the NIMH between 1981 and 1985 (mean follow-up interval = 8.8 years) revealed that only 41% of patients continued regular use of LT [10]. When queried as to why they discontinued using LT, perceived “ineffectiveness” and “inconvenience” were the two most commonly cited reasons. In the only clinical trial to include a planned, prospective follow-up of treated SAD participants during the next fall/winter season [11], only 4 out of 36 patients (11%) who received LT in the trial reported any use of LT during the next winter, and only 2 of those 4 reported using LT at a frequency, duration, and treatment length that would be expected to confer therapeutic benefits [7].

Therefore, as many as 47% of individuals with SAD do not remit with LT in the initial winter [9], and a minority of those treated acutely with LT continue using it over subsequent winters [10,11]. Extrapolating these data to the US prevalence of the disorder, over 11 million Americans could potentially benefit from the development of supplementary or alternative treatments, including 6.8 million that are projected to be non-responders to LT plus another 4.5 million that are projected to be LT responders, but will stop using the treatment. Ideal alternative treatments would be time-limited (i.e., acute treatment completed in a discrete period vs. daily treatment every fall/winter indefinitely), easier to implement in the long run relative to 5 months of daily LT each year, and have more durable effects than LT (i.e., effects that endure beyond the cessation of acute treatment to prevent annual depression recurrence).

Cognitive therapy for depression [12] is a time-limited psychotherapy treatment that appears to confer benefits that extend beyond the point of treatment termination [13,14]. Several studies have found that depressed patients who demonstrated a clinical response to cognitive therapy had a reduced risk of depression relapse as compared to patients who initially responded to antidepressant medications [15-18]. In addition to reducing the more proximal risk of relapse, one trial found that patients who had recovered from the treated episode with cognitive therapy demonstrated a reduced risk for a wholly new depressive episode onset (i.e., recurrence) relative to patients who had recovered from the initial episode with pharmacotherapy [16]. If these long-term outcomes for cognitive therapy generalize to SAD, the treatment could represent a more effective, practical, and palatable approach to long-term SAD management than LT. The integrative, cognitive-behavioral model of SAD [19] proposes a psychological vulnerability in SAD onset and maintenance, involving negative thinking biases, rumination, and disengagement from potentially enjoyable activities, which can be targeted by a SAD-tailored cognitive-behavioral therapy (CBT).

The primary aim of this NIMH-funded R01-level randomized clinical trial is to test the efficacy of CBT against LT in a larger, more definitive randomized head-to-head comparison on next winter outcomes in an intent-to-treat (ITT) analysis using all randomized participants. This project is focused on long-term depression outcomes in SAD. The primary aim tests for a clinically meaningful difference between CBT and LT on depression recurrence in the next winter (the primary outcome). A second annual winter follow-up will obtain preliminary data on the comparative effects of CBT vs. LT two winters after the initial winter of study treatment. In characterizing the course of major depressive disorder, the term “recurrence” refers to the onset of a wholly new major depressive episode occurring after recovery (i.e., full remission with minimal symptoms) from a previous episode [20]. The central public health challenge in the management of SAD is prevention of winter depression recurrence. Therefore, clinical practice will be impacted if prior CBT is associated with fewer SAD recurrences than initial treatment with LT.

### Pilot and feasibility studies

Prior to the commencement of this randomized clinical trial (RCT), Rohan and colleagues developed a novel, SAD-tailored, group CBT intervention and pilot tested it in an initial feasibility trial [21] and a controlled RCT [22]. This group subsequently published a follow-up report on next winter outcomes from the patients in these studies [11]. These studies were conducted with community adults in the greater Washington, D.C., area at the Uniformed Services University of Health Sciences in Bethesda, MD. In the feasibility study [21], 26 adults with SAD were randomly assigned to one of three 6-week treatments: CBT, light therapy (LT), or the combination of CBT and LT together (CBT+LT). Participants, in general, improved over all three treatments with large pre- to post-treatment changes in depression severity on blind interviewer- and patient-rated measures. The trial showed promise for the utility of CBT in acute SAD treatment, but was limited by the small sample size and lack of a control group. Therefore, the next study was a pilot controlled RCT [22] to better rule-out alternate explanations for treatment effects such as the passage of time or regression to the mean. The study maintained the three active treatment cells, but added a concurrent wait-list control group (WLC; 6 weeks of weekly symptom monitoring followed by treatment with LT) to the design. In the ITT sample of 61 randomized participants, all three active treatments (1) had significantly lower depression severity at post-treatment than WLC across continuous outcomes and (2) significantly improved over the 6 weeks, whereas WLC did not. Only CBT+LT (73%) had a significantly higher proportion of remissions than WLC (20%) at treatment endpoint.

Participants who were randomized to CBT, LT, or CBT+LT and who had not dropped out of the study by the end of acute treatment phase were invited to return to the laboratory for a naturalistic follow-up visit during the subsequent winter season (i.e., in January or February when SAD symptoms are typically at their worst). The follow-up report [11] was an ITT analysis of all randomized participants based on multiple imputation to estimate next winter outcomes for the 17 randomized individuals who dropped out during treatment, were withdrawn from protocol, or were lost to follow-up. The CBT (7.0%) and CBT+LT (5.5%) groups had significantly smaller proportions of winter depression recurrences than the LT group (36.7%). Solo CBT, but not combination treatment, was also associated with significantly lower interviewer- and patient-rated depression severity the next winter as compared to solo LT. Among completers who provided follow-up data, all statistically significant differences between the solo CBT and LT groups persisted after adjustment for ongoing treatment with LT, antidepressants, and psychotherapy. In contrast, CBT+LT and LT groups did not differ on next winter outcomes after adjustment for ongoing treatments. Therefore, despite having the highest post-treatment remission rate, initial treatment with combined CBT+LT did not appear to show the same lasting benefit over solo LT treatment on next winter outcomes as initial treatment with solo CBT. Observed differences between solo CBT and solo LT the next winter (i.e., a 30% difference in depression recurrence and 6- to 7-point differences in continuous depression scores) were large, clinically meaningful, and apparently not secondary to ongoing treatment use. Therefore, these findings require replication in a larger, more definitive study to determine whether CBT represents a more effective, practical approach to long-term SAD management than LT.

### Study aims

The primary aim is to compare the efficacy of CBT and LT on depression recurrence status, symptom severity, and remission status during the next winter season (i.e., the next wholly new winter season after the initial winter of treatment), which we argue to be the most important time point for evaluating clinical outcomes following SAD intervention. The hypothesis is that CBT will be associated with a smaller proportion of depression recurrences, less severe symptoms, and a higher proportion of remissions than LT in the next winter. The second aim is to compare the efficacy of CBT and LT on symptom severity and remission status at post-treatment (treatment endpoint). It is hypothesized that CBT and LT will not differ on post-treatment outcomes. An exploratory aim is to compare CBT and LT on recurrence, symptom severity, and remission two winters after treatment completion.

## Methods/design

### Design overview

This research was approved (IRB #08-116) by the Committee on Human Research in the Behavioral Sciences in the Research Protections Office at the University of Vermont, the site where the work is being conducted. This is a concurrent two-arm study that randomizes individuals experiencing a current SAD-related depressive episode to 6 weeks of treatment with group cognitive-behavioral therapy (CBT) or light therapy (LT). The primary endpoint is depression recurrence in the next winter, testing for two-sided differences in depression recurrence in one treatment condition relative to the other in an intention-to-treat (ITT) analysis. The proposed trial goes beyond prior studies in four ways. (1) This study augments the generalizability of prior data by relaxing the inclusion/exclusion criteria to allow for comorbid diagnoses and stable antidepressant medication use and by demonstrating the feasibility of training experienced community therapists to facilitate the CBT groups. (2) Recurrences and potential intervening variables that could affect outcome (e.g., new treatments, summer remission status) are prospectively tracked in the interim between treatment endpoint and the following winter. (3) This study includes a second annual winter follow-up to obtain preliminary data on the comparative effects of CBT vs. LT two winters after the initial winter of study treatment. (4) The influence of potential modifiers on the effects of CBT vs. LT will be examined, including demographic variables, baseline depression severity, comorbidity, baseline antidepressant medication status, and complete or incomplete summer remission status.

### Sample size and statistical power

This RCT is designed to definitively test for a significant and clinically meaningful difference between CBT and LT on depressive episode recurrence during the next winter season (the primary endpoint). The true difference between CBT and LT (and the difference we observe in this more rigorous design) may be smaller than what was observed in the above pilot study. To determine sample size, the more conservative estimate of a 23% difference in recurrence was used, based on a worst case scenario assumption that all subjects who dropped out of the pilot studies at any time from randomization through the next winter follow-up would have evidenced a recurrence the next winter (52% in LT vs. 29% in CBT in the ITT sample). If the true difference is 23%, 80 participants per group (total *N* = 160) are required to provide 80% power to detect a significant difference in recurrence between CBT and LT with a 2-sided test and alpha = 0.05. With 80 participants per group, there is also 80% power to detect a 5-point difference in next winter continuous depression scores based on variance estimates from a worst-case scenario analysis of pilot data whereby pre-treatment scores were substituted for any missing next winter scores (14.3 ± 11.5 for CBT vs. 19.5 ± 11.5 for LT on the Structured Interview Guide for the Hamilton Rating Scale for Depression—Seasonal Affective Disorder Version).

### Participants: inclusion and exclusion criteria

Adult community volunteers, aged 18 or older, are recruited for this study via local newspaper and radio advertisements and referrals from local health clinics beginning in September of each year. Inclusion criteria are: (a) DSM-IV-TR criteria for major depression, recurrent, with seasonal pattern, (b) Structured Interview Guide for the Hamilton Rating Scale for Depression—Seasonal Affective Disorder Version (SIGH-SAD) criteria for a current SAD episode (see below), and (c) no usage or stable use of antidepressants (i.e., a consistent dose of the same medication maintained for at least the past 4 weeks with no plans to change the medication or dose). Exclusion criteria include: (a) current LT or psychotherapy for depression or plans to initiate such treatment during the winter of study, (b) past LT or CBT for SAD, (c) presence of a comorbid axis I disorder that requires immediate treatment, (d) acute and serious suicidal intent, (e) positive laboratory findings for hypothyroidism at medical workup, and (f) plans for extended (>1 week) vacations or absences from the area through the coming March. Regarding the comorbidity exclusion (c), decisions are made on a case-by-case basis, but psychotic disorders, substance abuse/dependence, and bipolar-type SAD are uniformly excluded. Our criteria strike a balance between internal and external validity in ensuring that eligible volunteers receive a different experience in our CBT or LT conditions vs. a repetition of a past treatment while including a sample that generalizes to real-world SAD patients in terms of comorbidity and pharmacotherapy.

### Screening and enrollment

Given SAD’s inherent seasonal pattern, recruitment for this study begins each September and continues through mid-January. Individuals who respond to advertisements undergo a brief phone screening to assess inclusion/exclusion criteria. Respondents who do not endorse DSM-IV criteria for major depression, recurrent, with seasonal pattern and/or who satisfy any exclusion criterion on the phone screen receive a referral list. Respondents who appear to meet criteria on the phone screen are invited to review the informed consent document. We obtain informed consent before proceeding with any further study procedures. If they consent, the full Structured Clinical Interview for DSM-IV Axis I Disorders—Clinician Version (SCID-CV) is administered at an in-person screening visit. If a diagnosis of major depressive disorder, recurrent, with seasonal pattern is confirmed on the SCID and no axis I disorder requiring more immediate treatment is revealed, the Structured Interview Guide for the Hamilton Rating Scale for Depression—Seasonal Affective Disorder Version (SIGH-SAD) is administered to assess SIGH-SAD criteria for a current SAD episode. If SIGH-SAD criteria are not met, the individual is invited to attend SIGH-SAD interviews every other week. When a potential participant meets SIGH-SAD criteria for a current SAD episode, he or she is asked to attend a medical workup involving a routine physical examination and medical history to characterize the sample and assess the hypothyroidism exclusion. These evaluations are performed at the University of Vermont Clinical Research Center in the College of Medicine by a Physician’s Assistant. A nurse performs venipuncture to measure levels of TSH and thyroxine to screen for hypothyroidism. Individuals with laboratory results suggestive of hypothyroidism are excluded and referred for treatment. If the medical workup suggests normal thyroid functioning, the participant is asked to complete the pre-treatment assessment, at the conclusion of which he or she is randomized to a treatment group and enrolled in the study. At the end of January, SIGH-SADs are discontinued for any potential participant who did not meet the threshold for a SAD episode because of the low likelihood of developing a full threshold episode in that winter.

### Randomization procedure

Participants are randomly allocated to one of the two equal-probability, parallel, concurrent study treatments on a continuous basis as they qualify for the study. The schedule was designed by the project biostatistician/co-investigator (PMV) and was reviewed by an independent statistician prior to initiating the study. It was based on permuted random-size blocks of four and six, stratified by three variables: (1) sex, (2) major comorbid axis I diagnosis with two levels (present/absent), and (3) current antidepressant medication status with two levels (on medication or not). The randomization schedule is not available to anyone except the project statistician. Only the project coordinator, who does not conduct any assessments related to treatment outcome, is permitted to randomize participants.

### Treatments

#### Timing of the treatments

Participants enter the 6-week treatment phase on a continuous basis following randomization. The first week of February is the last treatment start date each fall/winter season of recruitment to ensure that all treatment is completed during the winter season and before spontaneous springtime remission typically occurs.

#### Light therapy (LT)

Participants randomized to LT attend an instructional session, standardized using a pre-generated script of points to cover: the LT treatment rationale, demonstration of assembly and positioning of the light box, prescription for daily use (i.e., timing and duration), possible side effects, and explanation of the daily LT self-monitoring diary and the side effects questionnaire. These sessions are audiotaped to assess treatment integrity. The PI conducts the sessions because the PI and the project coordinator are the only un-blinded personnel who work directly with participants, and the PI is qualified to answer any questions that arise.

LT participants use the SunRay (SunBox Company, Gaithersburg, MD), a standard LT unit (23 × 15½ × 3¼ inches) with an ultraviolet shield that emits 10,000 lux of white fluorescent light to the retina when the user is within 18 inches. Participants keep daily LT compliance diaries to record the timing and duration of LT. For each of the 6 treatment weeks, LT participants complete an LT side effects questionnaire to assess the presence and severity of any side effects attributed to LT (e.g., headache, eyestrain, feeling “wired) and self-reported times for onset of sleepiness, onset/offset of actual sleep, and desired times for onset/offset of sleep over the previous week. Although individuals typically respond within 4 weeks of initiating LT or not at all [7], our protocol is maintained and monitored for a full 6 weeks to match the duration of the CBT condition. To circumvent ethical concerns regarding discontinuation of a potentially beneficial treatment during the winter when relapse is likely [1], participants can choose to continue using the light box through April and are asked to return it in May. However, in an effort to reduce access to a suitable LT unit as a potential confound, LT participants who wish to use LT in the next fall/winter season are offered access to our light boxes if they agree to consult a provider for side effects management if needed. Continuing ongoing clinical management of LT under our supervision the next winter would create a potentially important confound as we do not offer to provide continued CBT or regular contact for CBT-treated participants. The chosen strategy strikes a balance between research design issues and ethical concerns related to patient safety.

Each week while LT is being administered, the PI calls the LT expert and co-investigator (TTP) to review weekly SIGH-SAD scores and responses on the side effects questionnaire. Because morning light is a more potent antidepressant than evening light as shown by direct comparisons [9,23] and because it has been suggested that morning light manifests its effects via correcting a pathological circadian phase-delay in SAD patients [24-26], LT is initiated at 30 min in the morning, first thing upon awakening, to be completed between the hours of 0600 and 0900 (i.e., in the phase advancing portion of the phase response curve to light; [27]). After the first week, Dr. Postolache recommends individually tailored, clinical adjustments to the duration of light use to maximize response and reduce any reported side effects. After each phone consult, the PI calls any LT participants with a recommended change to their LT prescription to convey the change. Increments in the duration of LT are initiated in response to an insufficient response to light, defined as a < 30% reduction in SIGH-SAD at the end of week 1, < 50% reduction in SIGH-SAD at the end of week 2, or not fulfilling SIGH-SAD remission criteria at the end of week 3 and beyond. In these cases, if the side effect profile permits, the duration of LT is increased in steps of 15 min/day up to a maximum of 2 h/day. Decrements in LT are based on side effects and consist of decreases in LT in 15-min increments/day. LT is temporarily halted for severe side effects (e.g., severe migraines) and restarted the following day with a reduced amount (50%), which is slowly increased to tolerance. There are two concerns related to circadian phase shifting induced by light: side effects of the phase shift and reduced antidepressant effectiveness if addressing the phase shift. In a recent re-analysis of Eastman et al.’s [28] data, evening light treatment for SAD phase delayed and morning light treatment phase advanced the temperature minimum (Tmin) by 1 h [29]. Early awakenings and/or late afternoon or early evening sleepiness sometimes reported by patients could be the result of a circadian phase advance induced by the morning administration of LT. If this occurs, the duration of the morning LT is reduced (if side effects present) and/or adjunct evening light is added.

#### Cognitive-behavioral therapy (CBT)

The PI tailored traditional cognitive therapy for depression [12] to the SAD population in developing the CBT for SAD protocol and its manual [30]. The treatment rationale addresses the role of environmental changes as well as cognitions and behavior in the onset and maintenance of seasonal depressive symptoms. CBT for SAD focuses on using traditional cognitive therapy elements of behavioral activation and cognitive restructuring to improve coping with winter. Behavioral activation is presented as a means to experience enjoyment during the fall/winter months by engaging in pleasurable activities. In addition to traditional cognitive restructuring of depressogenic thoughts, this treatment also identifies and challenges negative thoughts related to the winter season, low light availability (i.e., short days, cloud cover), and winter weather (e.g., cold temperatures, snow). To cope with subsequent winter seasons, each patient develops a personalized relapse-prevention plan, involving early identification of negative anticipatory thoughts about winter and of SAD-related behavior changes that occur early in his/her episode and using the skills learned to circumvent these prodromal signs of a SAD episode to prevent relapse and recurrence. Although cognitive therapy for depression is typically administered for 20 standard length (50-min) sessions over 16 weeks, SAD necessitates an intensified version. With winter lasting just 3 months, SAD patients may spontaneously remit with the arrival of spring if sessions were to be conducted over 16 weeks. Therefore, the protocol includes 12 longer (1½-h) sessions at a higher frequency (twice a week) over 6 weeks. Sessions are run in a small closed-group format with 4–8 participants per CBT group. Each group is led by a licensed PhD-level psychologist with a clinical graduate student co-therapist. All sessions are audiotaped to assess treatment adherence.

#### Therapist training/supervision

In addition to the PI therapist, two experienced (≥ 4 years of practice beyond training at study outset) doctoral-level community therapists facilitate the CBT groups, supervised by the PI. Study therapists were recruited who had clinical experience with depression and experience in delivering CBT treatments, were able to commit to the study, and were willing to learn the study treatment. To train the therapists in the CBT for SAD protocol, the PI conducted formal training sessions with the two therapists together to review the session-by-session manual content and to review and discuss audiotapes of past CBT for SAD group sessions. During the first CBT group that each study therapist led, the PI reviewed audiotapes of all 12 sessions as they occurred. Supervision during the first CBT for SAD group consisted of 1.5-hour weekly meetings between the PI and the group of therapists and graduate student co-therapists involving a combination of (1) group discussion of general issues in therapy, participant progress, and the treatment protocol and (2) individual time where the PI provided detailed feedback on the prior two sessions to each therapist/co-therapist dyad. After each therapist completed the first intensely supervised CBT group, the PI provided continued supervision each year while CBT groups were ongoing by holding weekly 1-hour meetings with the group of therapists and co-therapists to discuss the sessions that occurred that week. The PI continued to listen to a sample (20%) of all CBT sessions conducted to monitor therapist adherence to the protocol and to offer detailed feedback on a subset of sessions. Study therapists are compensated for their time in training and attending supervision at the going rate for a clinical hour in VT and for their time in administering CBT at the going per patient group therapy rate in VT.

### Treatment integrity

Because there were no pre-existing adherence/fidelity measures for CBT for SAD, the NIMH Collaborative Study Psychotherapy Rating Scale (CSPRS; Steven D. Hollon, PhD, personal communication, September, 2001), which measures the extent of specific therapist behaviors, was modified to measure adherence to our treatment protocols. CSPRS scoring procedures have been manualized [31], and adequate psychometric properties have been documented [32,33]. Original CSPRS items assessing specific CBT intervention components and nonspecific therapy aspects were retained with added items to assess psychoeducational and relapse-prevention components specific to our CBT manual. The language was modified to reflect group versus individual therapy. Items pertaining to clinical management were rephrased to reflect LT (as opposed to imipramine). A random sample (25%) of tapes is selected from each condition (CBT or LT) and session number and study therapist (in the case of CBT). Two trained clinical graduate students, blind to condition and session number, independently rate selected tapes. Inter-rater reliability and success at discriminating between the content of the CBT and LT conditions and between individual session numbers within the CBT condition will be computed. Patient’s treatment compliance is monitored by attendance and homework completion (for CBT) and LT diaries (for LT).

### Standardized information about ongoing and alternative treatments after study treatment ends

As a step to increase the likelihood that treatment in the interim is consistent with initial study treatment, standardized procedures are used for instructing participants with regard to treatment alternatives at the end of study treatment. When the treatment phase is complete in each study year, all participants receive a standardized letter, signed by the PI, encouraging them to keep up their study treatment and to pursue new treatments only if needed. Specifically, LT-treated participants receive a standard letter encouraging them to re-initiate daily LT upon onset of the first depressive symptom in the next fall/winter. The letter reiterates that LT participants can borrow our light boxes to use the next year as long as they have access to a professional to whom they can turn in the event of side effects and also lists contact information for LT manufacturers should they wish to purchase their own unit. CBT-treated participants receive a standard letter encouraging them to keep using the skills learned in the CBT for SAD treatment on their own. Both letters include the same, standardized safety net in providing contact information for local mental health centers and treatment providers should they require more formal treatment. This approach takes into account the ethical concern of not discouraging participants from getting needed treatment between post-treatment and the next winter follow-up while recognizing that we cannot ethically proscribe treatment and are accountable for directing them to resources.

### Formal follow-up assessments

Three formal, in-person follow-up assessments occur subsequent to study treatment completion: in the summer (i.e., the subsequent August), the next winter (i.e., January or February of the next wholly new winter season), and the second winter (i.e., January or February of the second wholly new winter season). All randomized participants are invited to attend follow-ups.

### Phone tracking of recurrences and retreatment

A blinded clinical graduate student telephones each participant twice in the interim between summer follow-up and the in-person next winter follow-up: once in October and once in December. The purpose of these brief phone contacts is to track any recurrences and any new treatments initiated. To track recurrences, DSM-IV-TR criteria for a major depressive episode on the SCID-CV are assessed for the interval between the present and last contact (i.e., formal assessment or phone call). To track retreatment, the caller asks, “Have you started any new treatments since (date of last contact) such as LT, talk therapy, or medications?” If the participant answers affirmatively, he or she is queried for specific details.

### Outcome measures

#### Structured Interview Guide for the Hamilton Rating Scale for Depression—Seasonal Affective Disorder Version (SIGH-SAD)

The SIGH-SAD [34] includes the 21-item Structured Interview Guide for the Hamilton Rating Scale for Depression (HAM-D) and a supplementary 8-item subscale to assess atypical depressive symptoms associated with SAD. A trained rater, blind to the treatment condition, administers the SIGH-SAD at pre-treatment, treatment weeks 1–5, post-treatment, summer follow-up, next winter follow-up, and second winter follow-up. A second blinded rater subsequently rates audiotapes of the live SIGH-SADs to assess inter-rater reliability.

The primary outcome, depression recurrence status in the next winter, is defined according to the following SIGH-SAD criteria [35]: total SIGH-SAD score ≥ 20 + HAM-D score ≥ 10 + atypical score ≥ 5. The same criteria are used to define SAD episode onset (i.e., study inclusion criterion b) and depression recurrence status at follow-ups. Continuous depression scores and remission status on the SIGH-SAD represent secondary outcome measures. One or both of the following SIGH-SAD criteria are used to classify a full remission at post-treatment [36]: pre- to post-treatment reduction in total SIGH-SAD score ≥ 50% + HAM-D score ≤ 7 + atypical score < 7 OR HAM-D score ≤ 2 + atypical score ≤ 10. These remission criteria are also used to define remission status at the summer follow-up and annual winter follow-ups, except that the respective follow-up SIGH-SAD score, as opposed to the post-treatment score, is contrasted to baseline status.

#### The Longitudinal Interval Follow-up Evaluation (LIFE)

The LIFE [36] is a semi-structured interview that assesses the longitudinal course of psychiatric disorders, including remissions and recurrences. This study uses the psychopathology section of the LIFE, which provides weekly depression status ratings on a continuous symptom scale ranging from 1 (usual self; no residual depressive symptoms) to 6 (definite DSM-IV-TR Major Depressive Episode criteria and extreme impairment), to assess depression in the following three interim periods: between post-treatment and the subsequent summer follow-up, between summer follow-up and the next winter follow-up, and between the next winter follow-up and the second winter follow-up. Any 2-week or longer period of depression ratings in the range of 5 (the threshold for meeting DSM-IV-TR Major Depressive Episode criteria) or 6 will be used as a secondary outcome of depression recurrences that occur between assessments. The LIFE was used in this way in a 2-year follow-up of depressed patients treated with cognitive therapy or antidepressant medications to capture relapse and recurrence [16,37]. A trained and blinded clinical graduate student rater administers the LIFE live, and a second blinded clinical graduate student rates an audiotape of the LIFE to assess reliability for detecting recurrences in these interim periods.

### Beck Depression Inventory—Second Edition (BDI-II)

The BDI-II [38] is a 21-item measure of depressive symptom severity that constitutes a secondary outcome measure, administered at pre- and post-treatment, summer, next winter, and the second winter. The BDI-II has demonstrated good test-retest reliability and convergent validity [38]. Consistent with the pilot trials [11,21,22], BDI-II ≤ 8 is a secondary measure of remission status.

### Protocol for training clinical raters and ensuring accuracy of scores

To train SIGH-SAD raters, each year of recruitment, the PI leads several sessions with the study team to discuss each of the 29 SIGH-SAD items in detail and the nuances of scoring them. Subsequently, each year, the raters practiced rating at least three audiotapes of SIGH-SADs from past studies and discussed their ratings in a group session lead by the PI. Trainees were required to observe a veteran rater perform a live SIGH-SAD in the study, record their ratings, and discuss them with the interviewer after the session. To become an independent rater, a trainee had to perform a mock SIGH-SAD interview on the PI (role-playing a patient) with good flow and proficiency and obtain item ratings that corresponded well to the PI’s judgments.

The following protocol was developed to increase the accuracy of SIGH-SAD scores. The project statistician identifies any SIGH-SAD administration with a 5-point or greater discrepancy in total score (minus items H16 and H17, which require a direct observation of the patient) between the original and second rater. In these cases reaching the threshold for an unacceptable split between raters, two additional blinded raters are selected from our team to re-rate those particular interviews. The statistician then examines ratings from all four raters of each tape to identify SIGH-SAD items where the original rater disagreed with two or three of the other three raters. Subsequently, the two new raters discuss those particular SIGH-SAD items and try to reach a consensus on the most accurate score for them. If a consensus cannot be reached, the PI is consulted about what was said on the tape (keeping her blind to subject identity, and, therefore, group assignment) to make a final decision about the most accurate score for that item. Any corrected SIGH-SAD scores will be used in the primary and secondary analyses. However, inter-rater reliability statistics will still reflect agreement between the original (uncorrected) rater and the second rater.

### Data analysis plan

The primary analysis is an intent-to-treat (ITT) analysis based on multiple imputation (MI) of missing next winter SIGH-SAD scores [39-41]. Imputed scores will be used to classify depression recurrence status for individuals who dropped out during the treatment phase, were withdrawn from protocol, or were subsequently lost to follow-up. Imputed values will be generated from multivariate normal models derived from subjects with next winter follow-up data, using baseline and post-treatment SIGH-SAD, HAM-D, and atypical scores, demographic variables (e.g., gender, race, age), and other baseline patient characteristics (e.g., presence of a comorbid axis I disorder, antidepressant medication status) as potential predictors. Separate models will be developed for the CBT and LT groups rather than including treatment group in the model as a predictor, because in the pilot study, baseline SIGH-SAD scores were negatively correlated with next winter scores in CBT and were positively correlated with next winter scores in LT. Outcomes for subjects with missing next winter data will be predicted from the models based on available data and a random component reflecting the residual distribution for the dependent variable. Ten data sets with different imputed values will be generated, and a dichotomous variable indicating recurrence status will be computed for each subject based on SIGH-SAD criteria. The difference between the CBT and LT groups in the proportions of subjects with a recurrence the next winter will be estimated for each of the ten data sets and the estimates will be combined using the inference methods for MI described by Little and Rubin [42]. SAS PROC MI and PROC MIANALYZE will be used to carry out the imputation and analysis. Sensitivity analysis will be conducted to determine the robustness of the results under alternative MI models and imputation methods, including best and worst case scenarios for each treatment.

The above analysis will be repeated using the following alternate recurrence definitions as secondary outcomes: (1) meeting SIGH-SAD recurrence criteria at the prospective next winter follow-up OR depression recurrence defined as any major depressive episode as assessed by the LIFE OR fulfilling DSM-IV-TR major depression criteria in the interim between summer and next winter as assessed by the October and December tracking telephone calls, (2) a recurrence on the next winter SIGH-SAD OR depression recurrence status as assessed by considering any new treatment reported in the interim between treatment endpoint and next winter follow-up (except for ongoing LT for LT participants and using CBT skills on their own without a therapist for CBT participants) as prima facie evidence of recurrence, and (3) the most encompassing and conservative definition of recurrence that defines a recurrence as fulfilling criteria (1) OR (2) above.

Secondary analyses will examine how potential modifiers influence the effects of CBT and LT on the primary outcome of next winter recurrence on the SIGH-SAD. Potential modifiers will include: (1) demographic variables such as sex, age, and race (White vs. minority); (2) baseline characteristics such as pre-treatment depression scores, axis I comorbidity, and baseline antidepressant medication status; and (3) complete or incomplete summer remission status in the interim, as ascertained by the SIGH-SAD at the summer follow-up. This will be done using logistic and linear regression analyses for dichotomous and continuous outcomes, respectively, and including the modifiers as well as their interactions with treatment in the models. Although the models are essentially the same as those fitted to the complete data for the purpose of imputation, for this analysis they will be fitted to the imputed data sets, and the Rao-Blackwell estimates of the effects of the modifiers will be obtained [43].

## Discussion

The study design was selected to answer the primary question: Do CBT and LT differ on depression recurrences during the subsequent winter season? The simple, straightforward CBT vs. LT design will answer that question and logically stems from pilot study findings. As a time-limited treatment, if CBT is associated with better outcomes than LT during the subsequent winter, CBT may represent a more practical, effective approach to long-term SAD management in clinical practice. The unique methodological challenges faced here likely generalize to designing intervention trials for other predictably recurrent and time-limited problems focused on long-term outcomes. Justification for the chosen design, consideration of alternative approaches, challenges faced with this population, and areas for future study are discussed below. Alternative designs would have required more patients, increased costs, and reduced power to detect a difference in the primary outcome.

### Rationale for not designing to detect differences between CBT and LT at treatment endpoint

The pilot study findings suggest that differences between CBT and LT following 6 weeks of treatment are quite small and that powering studies to detect these differences would not represent cost-effective use of resources. For example, 350 participants would be required in each treatment arm (*N* = 700) for a 2-group Χ^2^ test with a two-sided 0.05 significance level to achieve 80% power to detect the 10% difference in proportions remitted on the SIGH-SAD at post-treatment of 56% in LT vs. 46% in CBT (observed rates pooling across data from Rohan et al., 2004, 2007). Moreover, the effect size associated with this 10% difference in remission is small, h = 0.20, and its meaning is not clear in terms of cost-benefit. Similarly, post-treatment differences between CBT and LT on continuous depression scores were consistently less than 2 points, a difference that is quite small and may not be clinically meaningful. The central public health challenge with regard to SAD is prevention of episode recurrence over subsequent winter seasons. Therefore, the current trial focuses on detecting a significant and clinically meaningful difference in the proportion of depressive episode recurrences between CBT and LT at the next winter follow-up if this difference exists.

### Rationale for not designing to establish equivalence between CBT and LT the next winter

The motivation for this trial is based on pilot data showing that depression recurrence in the next winter season may be substantially lower with CBT than with LT [11]. Therefore, it is more important to test whether depression recurrence in the subsequent winter is lower in one treatment relative to the other than to test for equivalence between CBT and LT. If CBT and LT are “equivalent” (i.e., the true difference between the therapies is functionally equivalent to zero, indicating the absence of a difference between the treatments), it would require 280 subjects in each cell (*N* = 560) to test if recurrence rates are equivalent within 10% (i.e., LT-CBT > −0.10). An equivalence study of this size is not feasible or warranted in light of the pilot data. Furthermore, a reduction of recurrence would be the most, if not the only, compelling reason for widespread adoption of CBT in clinical practice because the vast majority of practitioners and researchers who specialize in SAD have a chronobiological orientation and enthusiasm for LT.

### Rationale for following all randomized participants vs. only treatment responders

Although the more common approach in the nonseasonal depression literature is to consider only the long-term outcome of acute treatment responders rather than that for all randomized participants, SAD patients are unique because, by definition, they should spontaneously remit in the summer, which is prospectively assessed in this study. In addition, focusing only on acute responders does not capture a potentially important clinical phenomenon whereby it may be easier for SAD patients to use CBT in the prevention of symptoms during the subsequent winter than in the treatment of an acute SAD episode in the initial winter. Therefore, we chose to follow all randomized participants.

### Rationale for using the established treatment (LT) as the control group

The lack of other types of control groups here will not allow determination of whether the treatments might be equally ineffective, rather than comparably effective, in the acute treatment of SAD. Instead, our primary aim addresses the concern that the treatments might be equally ineffective by testing for a meaningful difference between treatments on depression recurrence in the next winter. The addition of a wait-list control group is not ethically justified in this study because participants randomized to the wait-list would be asked to endure an untreated major depressive episode in the initial winter and also to endure the likely recurrence of their depression without treatment in the next winter season. In our judgment, withholding treatment for two consecutive fall/winter seasons would place these participants at significant risk, and these safety concerns substantially outweigh the possible scientific benefit to be gleaned from such a comparison.

### Rationale for not including an attentional control group

If the efficacy of CBT for SAD becomes firmly established, there are other valid questions that could be asked in future studies that would require a different type of control group. The present study does not address the question of whether nonspecific factors such as group processes, social support, therapist attention, etc.—as opposed to the cognitive and behavioral treatment components—are responsible for CBT’s observed treatment effects. Because our CBT for SAD protocol is a relatively new treatment, research designs that address its efficacy are more relevant than designs addressing its mechanisms at this point. If the pilot findings are replicated here and in future studies at other sites, dismantling studies to isolate and identify the active therapeutic components of our CBT will be indicated. Such pursuits are premature to date.

### Rationale for not including a combined CBT+LT treatment

In pilot studies [11,21,22], CBT+LT was associated with the highest proportion of SIGH-SAD remissions at post-treatment, but did not appear to show as much lasting benefit as CBT alone over LT on next winter outcomes. Moreover, a descriptive comparison of SIGH-SAD and BDI-II remission rates at post-treatment to the next winter remission rates suggests that only solo CBT treatment continued to improve from post-treatment status into the subsequent winter season. In contrast, the remission rates observed for solo LT and CBT+LT treatment appeared to deteriorate somewhat from post-treatment to the next winter. The reasons behind this apparent decay in CBT+LT’s effects over the subsequent winter season are not known. It is possible that participants benefited from combined treatment in the initial winter because they fully engaged in both protocols under the study team’s close monitoring and supervision during the 6 weeks. During the subsequent winter season, however, CBT+LT participants had to make a decision about what treatment(s) to continue and how to balance the treatment components, which might have contributed to perceived burden, treatment overload, or attributing treatment effects to one treatment over the other. Initial treatment with CBT alone may lead to a more parsimonious course of action the next winter. Although we might continue to explore the CBT+LT combination and ways to augment its long-term effects in future studies, the current project will test for a superiority of one treatment over the other on depression recurrence in the next winter, which is the next logical step in the development and testing of CBT for SAD.

## Trial status

Recruitment for this trial has been ongoing since September 2008.

## Abbreviations

BDI-II: Beck Depression Inventory-Second Edition; CBT: Cognitive-behavioral therapy; CBT+LT: Cognitive-behavioral therapy combined with light therapy; CSPRS: Collaborative Study Psychotherapy Rating Scale; DC: District of Columbia; DSM-IV-TR: Diagnostic and Statistical Manual of Mental Disorders Fourth Edition-Text Revision; HAM-D: 21-item Structured Interview Guide for the Hamilton Rating Scale for Depression; H: Hours; ITT: Intent-to-treat; LIFE: Longitudinal Interval Follow-up Evaluation; LT: Light therapy; MD: Maryland; MI: Multiple imputation; MIN: Minutes; NIMH: National Institute of Mental Health; PI: Principal investigator; RCT: Randomized clinical trial; SAD: Seasonal affective disorder; SCID-CV: Structured Clinical Interview for DSM-IV Axis I Disorders—Clinician Version; SIGH-SAD: Structured Interview Guide for the Hamilton Rating Scale for Depression—Seasonal Affective Disorder Version; Tmin: Temperature minimum; TSH: Thyroid-stimulating hormone; VT: Vermont; WLC: Wait-list control.

## Competing interests

KJR receives book royalties from Oxford University Press for the treatment manual for the cognitive-behavioral therapy for SAD intervention (Rohan, 2008). The authors have no other financial or nonfinancial competing interests.

## Authors’ contributions

KJR conceptualized the project, serves as the project’s principal investigator, and led the team in manuscript preparation. ME, JNM, LS, SH, and YIN made substantial contributions to acquisition of data and were involved in critically revising the manuscript for intellectual content. TTP aided KJR in conceptualizing the project and wrote the sections of the manuscript pertaining to the light therapy intervention. PMV aided KJR in designing the project, serves as co-investigator/project biostatistician, and wrote the sections of the manuscript related to statistical power and data analysis. All authors read and approved the final manuscript.
